# Melatonin Alleviates Oxidative Stress Induced by H_2_O_2_ in Porcine Trophectoderm Cells

**DOI:** 10.3390/antiox11061047

**Published:** 2022-05-25

**Authors:** Yawei Fu, Yue Chen, Zhao Jin, Hu Gao, Gang Song, Qian Wang, Kang Xu

**Affiliations:** 1 College of Animal Science and Technology, Henan Agricultural University, Zhengzhou 450000, China; fuyw2020@163.com (Y.F.); 18837025618@126.com (Y.C.); 2 Institute of Subtropical Agriculture, Chinese Academy of Sciences, Changsha 410125, China; jz2725552543@163.com (Z.J.); gaohu_20190008@163.com (H.G.); sg19971109@163.com (G.S.); wangq0130@163.com (Q.W.)

**Keywords:** Placental, PTr2 cells, ROS, cell viability, apoptosis

## Abstract

Placental oxidative stress has been implicated as a main risk factor for placental dysfunction. Alleviation of oxidative stress and enhancement of antioxidant capacity of porcine trophectoderm (PTr2) cells are effective means to maintaining normal placental function. The present study was conducted to evaluate the protective effect of melatonin (MT) on H_2_O_2_-induced oxidative damage in PTr2 cells. Our data revealed that pretreatment with MT could significantly improve the decrease in cell viability induced by H_2_O_2_, and reduce intracellular reactive oxygen species (ROS) levels and the ratio of apoptotic cells. Here, we compared the transcriptomes of untreated versus melatonin-treated PTr2 cells by RNA-seq analysis and found that differentially expressed genes (DEGs) were highly enriched in the Wnt signaling, TGF-beta signaling and mTOR signaling pathways. Moreover, pretreatment with MT upregulated the antioxidant-related genes such as early growth response3 (EGR3), WAP four-disulfide core domain1 (WFDC1), heme oxygenase1 (HMOX1) and vimentin (VIM). These findings reveal that melatonin protects PTr2 cells from H_2_O_2_-induced oxidative stress damage.

## 1. Introduction

Pregnancy is particularly sensitive to oxidative stress, defined as increased basal oxygen consumption in both the fetus and the mother [1]. Numerous factors can trigger oxidative stress during gestation. The placenta plays an important role in maintaining fetal and maternal homeostasis, is rich in mitochondria and consumes approximately 1% of the basal metabolic rate of the mother when fully developed [2]. Meanwhile, the placental environment transforms from a hypoxic environment to an oxygen-rich environment as the placenta matures and its vascularization develops. Considering, the placenta’s significant mitochondrial mass, high metabolic rate and oxygen-rich environment, the generation of ROS increases in the placenta, which is easily subjected to a degree of oxidative stress.

During pregnancy, the placenta is susceptible to oxidative stress and has a reduced antioxidant capacity, which can pose a potential problem for late animal reproduction and affect maternal homeostasis and fetal growth and development [3,4,5]. PTr2 cells are a significant cell type of the placenta, and they represent one of the earliest events of spectral differentiation in mammalian embryos [6,7,8]. Placental oxidative stress is involved in pregnancy complications, and previous studies have shown that oxidative stress of PTr2 cells can lead to pathological conditions of pregnancy (early pregnancy loss and impaired placentation) [9,10]. Therefore, improving the defensive ability of PTr2 cells to effectively counteract ROS generation is beneficial to maintaining fetal pig growth and increasing the productive performance of dams.

The imbalance between ROS production and antioxidant capacity leads to oxidative stress [11]. To prevent oxidative stress, the supply of antioxidants is essential. Although endogenous antioxidants help protect against oxidative damage, alone they are insufficient to solve the problem. In preclinical studies, antioxidants (vitamins C and E) can lower maternal oxidative stress during pregnancy, but decreased fetal growth and abnormal maternal blood pressure might occur with a certain probability [12]. There are few satisfactory treatment strategies for these conditions, thus, developing new strategies to alleviate oxidative stress is of great significance.

MT is an endogenous substance with antioxidant properties [13]. MT exerts antioxidative action through multiple pathways [14]. On the one hand, MT protects the body from the harmful effects of free radical damage [15,16]. On the other hand, MT alleviates oxidative stress and protects the ovaries from oxidative damage by elevating antioxidant enzyme activity [17,18]. The antioxidant effects of MT can protect cells from the damage of oxidative stress damage [19,20]. However, its protective effects against PTr2 cells oxidative stress and the underlying antioxidant mechanism have yet to be investigated.

In this study, we investigated the protective effects of MT against H_2_O_2_-induced oxidative damage in PTr2 cells. Our findings can provide a theoretical basis for the development of the clinical application of MT during pregnancy and provide guidance for developing effective nutritional strategies to improve sow health.

## 2. Materials and Methods

### 2.1. Cell Culture and Sample Preparation

The PTr2 cell line used in this experiment were previously established and characterized from porcine blastocysts (collected on day 12 of pregnancy) [21,22]. Cell cultures were grown under completely sterile conditions and kept incubated at 37 °C in a 5% carbon dioxide (CO_2_) atmosphere. When the culture reached 80−90% confluence, cells were detached from the culture flasks by 0.25% trypsin treatment and counted, and the concentration was adjusted to 10^4^ cells/mL (0.1 mL) of DMEM/F12 complete medium for subsequent experiments.

Chemicals were purchased from Thermo Fisher Scientific (Fair Lawn, NJ, USA): 1% penicillin–streptomycin liquid (catalog #: 15140122); DMEM/Ham’s F-12 medium (DMEM/F12; catalog #: 11330032); 10% fetal bovine serum (FBS; catalog #: 10099141); and trypsin-EDTA (catalog #: 25200-056, purchased as 0.25%). Cell culture plates and plastic flasks were purchased from Corning Incorporated (Corning, NY, USA). MT was purchased from Sigma—Aldrich: (catalog #: M5250).

### 2.2. Evaluation of the Cell Protection against Oxidative Damage

To evaluate the protective effect against oxidative stress in PTr2 cells after treatment with MT, preliminary experiments were performed to determine assess cell viability and determine an appropriate concentration of H_2_O_2_ for subsequent experiments by the CCK-8 assay [23]. PTr2 cells were inoculated into 96-well plates, treated with 50, 100, 200 and 500 μM MT and cultured at 37 ℃ with 5% CO_2_ for 24 h. Then, the cells were stimulated with 120 μmol/L H_2_O_2_, and after 4 h, cell viability was measured by the CCK-8 assay [24]. The relative number of viable cells can be determined from the measured absorbance value (OD value). There were six replicate sets of wells per MT concentration.

Cell viability was calculated by the following formula:

Cell viability (%) = (treatment group OD − blank group OD)/(control group OD − blank group OD) × 100.

### 2.3. Apoptosis Detection

To further investigate whether MT could protect against H_2_O_2_-induced cell apoptosis in PTr2 cells, apoptosis rates were evaluated by flow cytometry using Annexin V-FITC/PI [25]. Based on the 2.2 testing, PTr2 cells were incubated in a 6-well plate (1 × 10^5^ cells/well) and pretreated with MT (0, 100, 250, 500 and 1000 μM) for 24 h, and subsequently treated with 120 μM H_2_O_2_ for 4 h. The cells were washed; 2 μg annexin-V FITC and 10 μL PI were added, and then the cells were incubated in the dark for 15 min and analyzed on a flow cytometer (ex488 nm and em578 nm, Becton Dickinson, San Jose, CA, USA).

### 2.4. Measurement of Intracellular ROS

The intracellular ROS levels were detected by flow cytometry using the ROS-specific fluorescence dichlorodihydrofluorescein diacetate (DCFH-DA) [26]. Based on the 2.2–2.3 testing, PTr2 cells were incubated in a 6-well plate and pretreated with MT (0, 250 and 500 μM) for 24 h, and subsequently treated with 120 μM H_2_O_2_ for 4 h. After that, cells washed twice with PBS, and then incubated with 10 μM DCFH-DA in PBS at 37 °C for 30 min. After the cells were collected and the fluorescence intensity of DCF was quantified on a FACS Calibur (λex/em = 488/525  nm). Then, mean intensity of ROS was collected from 1 × 10^5^ cell counts. Finally, the intracellular ROS levels and their analyses were performed using a FACS Calibur Flow Cytometer (BD Biosciences).

### 2.5. Transcriptome Sequencing

PTr2 cells were grouped based on reference to the transcriptome sequencing, and the cells were cultured in 6-well plates (1 × 10^5^ cells/well) with 500 μM MT and 120 μM H_2_O_2_ at the indicated time points, as described above. The PTr2 cell samples of three individuals (designed as biological replicates) in each of the four groups were subjected to high-throughput transcriptome sequencing, and the rest of the samples were reserved for standby using. Total mRNA was extracted from cells as previously described. RNA sequencing data were processed using Trimmomatic [27], gene expression calculation using cufflinks and the read counts of each gene were obtained by htseq-count [28,29], differentially expressed genes (DEGs) identification with the DESeq R package functions estimate size factors and the nbinom test [30], gene ontology (GO) enrichment analysis and pathway enrichment were performed using R based on the hypergeometric distribution [31,32]. The high-quality reads were aligned to the pig reference genome (UCSC susScr3) using Hisat2 software [33]. Then, the protein–protein interaction network (PPIs) information of these DEGs was predicted by the STRING database (version 11.5) [34]. After mapping the DEGs into this database, and a combined score ≥ 0.4 was exported [35]. Then, the PPIs of these DEGs were visualized in Cytoscape, and the hub genes among the PPI network were identified and ranked using the CytoHubba plugin and the maximal clique centrality (MCC) method of Cytoscape software (Version 3.8.2; The Cytoscape Consortium, New York, NY, USA).

The grouping information is as follows:

(1) Control group: The PTr2 cells were cultured in basic growth medium (DMEM,10% FBS) were used as a control;
(2) MT group: The PTr2 cells were treated for 24 h in a medium containing 500 μM MT;
(3) H_2_O_2_ group: The PTr2 cells were grown in normal medium for 24 h, washed with PBS three times and treated with H_2_O_2_ for 4 h;
(4) MT-H_2_O_2_ (MH) group: The PTr2 cells were grown in a medium containing 500 μM MT for 24 h, washed with PBS three times and treated with H_2_O_2_ for 4 h.

### 2.6. Real-Time PCR Analysis

To verify the data of RNA-Seq data, real-time quantitative PCR (qRT–PCR) was performed on six randomly selected DEGs. The *β-actin* gene was used as the reference gene, and the primers for the six genes were designed using Primer5. Real-time PCR was performed using the miScript SYBR Green PCR kit (Thermo) to detect the expression of DEGs on the Rocho Lightcycler 480II (Roche). We calculated the relative gene expression levels with the comparative CT method (referred to as the 2^−^^△△CT^ method), with three replicates for each reaction [36].

### 2.7. Western Blot Analysis

PTr2 cells were cultured in different media and performed as described in the methods. After two washes with PBS, the cells were lysed with protein extraction reagent and harvested (Thermo Fisher Scientific Inc., Waltham, MA, USA) [37]. The protein concentrations were determined by using a BCA kit. All protein samples were diluted to an equal concentration using RIPA buffer. Western blot analysis was performed as previously described with minor modifications [38]. After protein concentration determination, the extracted proteins were loaded on a 10% separation gel and 4% concentration gel, and then transferred onto PVDF membranes. The proteins were sealed with 5% skimmed milk powder, and then incubated overnight with the indicated primary antibodies at 4 °C, followed by incubation at room temperature for 2 h with secondary antibodies. Experiments were conducted using the following primary antibodies: *WFDC1* (1:500, OM117200, omnimAbs), *HMOX1* (1:500, 27282-1-AP, Proteintech), *MXI1* (1:500, OM105330, omnimAbs), *PLAG1* (1:500, OM108610, omnimAbs), *ViIM* (1:200, OM115585, SANTA CRUZ), *EGR3* (1:500, OM106724, omnimAbs) and *β-actin* (1:5000, 66009-1-Ig, Proteintech).

### 2.8. Statistical Analysis

The statistical analysis was performed using one-way ANOVA with SPSS software (version 18.0, USA), and values were expressed as the mean ± SEM (standard errors of means) where the *p*-value was considered less than 0.05 and it was statistically significant.

## 3. Results

### 3.1. Effects of H_2_O_2_ on the Viability of PTr2 Cells at Different MT Concentrations

**Figure 1:**
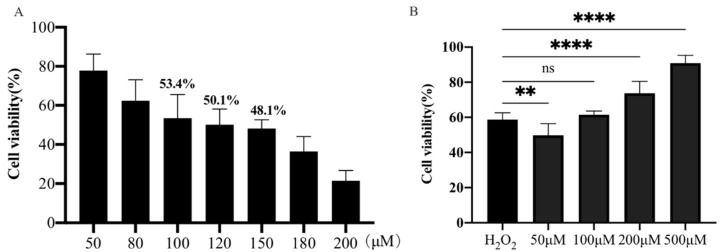
Effect of H_2_O_2_ on antioxidant properties of PTr2 cells. (**A**) The PTr2 cells were treated with different concentrations of H_2_O_2_ for 4 h, and then the cell vitality was detected using a CCK-8 assay. (**B**) The PTr2 Cells were cultured with different concentrations of MT for 24 h before incubated with 120 μM H_2_O_2_ for 4 h and cell viability was detected using a CCK-8 assay. The data are expressed as the means ± SEM from at least 6 separate experiments. *p* < 0.05 was considered to be statistically significant. *p* > 0.05 (ns), *p* < 0.01 (**), *p* < 0.0001 (****).

Initially, the viability of PTr2 cells treated with different concentrations of H_2_O_2_ was determined using a CCK-8 assay. As shown in Figure 1A, the 120 μM H_2_O_2_ was used to establish the oxidative stress model of PTr2 cells, and the cell viability decreased to 50.15 ± 0.03% compared with the control group after induction for 4 h. PTr2 cells were pretreated with different concentrations of MT (50, 100, 200 and 500 μM) for 24 h, and then treated with 120 μM H_2_O_2_ for 4 h. As shown in Figure 1B, pretreatment with 500 μM MT significantly enhanced the viability of H_2_O_2_-treated PTr2 cells.

### 3.2. Protective Effects of MT against H_2_O_2_-Induced Apoptosis in PTr2 Cells

**Figure 2:**
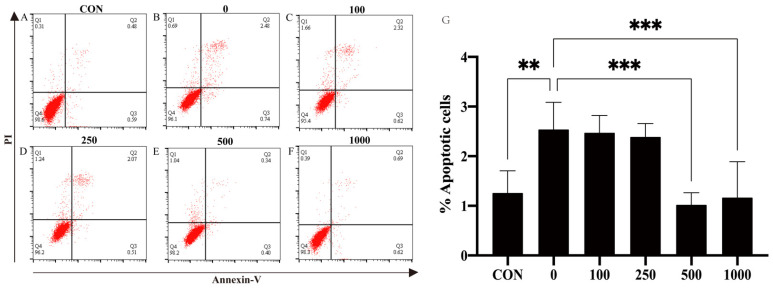
Effects of MT treatment on H_2_O_2_-induced apoptosis in PTr2 cells. The PTr2 cells were treated with medium as control (A) and MT at the concentrations of 0 (B), 100 (C), 250 (D), 500 (E), and 1000 (F) for 24 h and then exposed to H_2_O_2_ for 4 h. (G) Histograms showed that distribution of apoptotic cells after different treatment. The data are expressed as the means ± SEM from at least 6 separate experiments. *p* < 0.05 was considered to be statistically significant. *p* > 0.05 (ns), *p* < 0.01 (**), *p* < 0.0001 (***).

To further investigate whether MT could protect against H_2_O_2_-induced cell apoptosis in PTr2 cells, apoptosis rates were evaluated by flow cytometry using Annexin V-FITC/PI. Compared with the group, the cell apoptosis rate was significantly (*p* < 0.05) increased in the H_2_O_2_ group. The increased apoptosis rate of PTr2 cells caused by H_2_O_2_ induction was significantly (*p* < 0.05) decreased after MT pretreatment (Figure 2).

### 3.3. Effect of MT on H_2_O_2_-Induced ROS Production in PTr2 Cells

**Figure 3:**
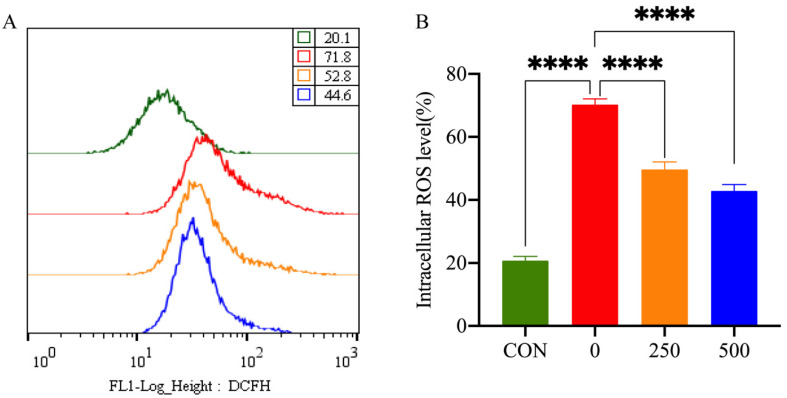
Effect of MT on H_2_O_2_-induced release of ROS in PTr2 cells. (**A**) Intracellular ROS level was detected by flow cytometry; (**B**) Histogram analysis showing the intracellular ROS levels. The data are expressed as the means ± SEM from at least 6 separate experiments. *p* < 0.05 was considered to be statistically significant. *p* > 0.05 (ns), *p* < 0.0001 (****).

To study the antioxidant activity of MT, the intracellular ROS levels of PTr2 cells were examined by measuring the DCFH fluorescence intensity (Figure 3). Treatment with H_2_O_2_ significantly increased the intracellular level of ROS in PTr2 cells. However, 500 μM MT pretreatment significantly reduced ROS production.

### 3.4. Transcript Expression in PTr2 Cells

**Figure 4:**
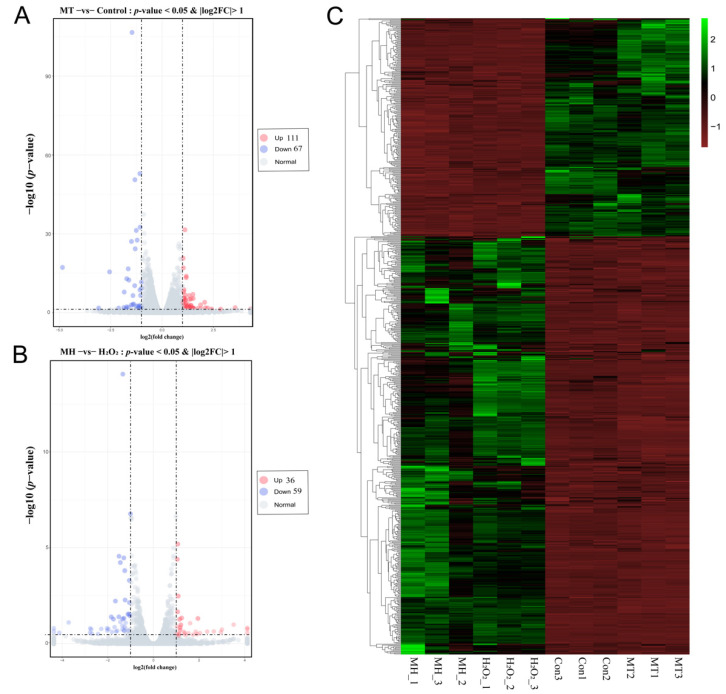
Effect of MT on mRNAs expression of PTr2 cells. (**A**) The volcano figure analysis of differentially expressed mRNAs in MT and control groups of porcine trophectoderm cells by RNA-seq (**B**) The volcano figure analysis of differentially expressed mRNAs in MH and H_2_O_2_ groups of PTr2 cells by RNA-seq. The pink, blue, and grey colors represent the terms means upregulation downregulation and normal expression, respectively. (**C**) Cluster analysis of DEGs by the FPKM value. The *x*-axis indicates the samples in the different groups. The *y*-axis is the gene cluster across different groups. Green indicates the highly expressed genes, and red indicates the genes with low expression by the value of log_10_ (FPKM + 1).

To investigate genes affected by MT, gene expression profiling of the control, MT, MH and H_2_O_2_ groups was performed: A total of 178 DEGs were obtained in MT group compared with the control group, including 67 downregulated and 111 upregulated genes (Figure 4A); Similarly, compared with the H_2_O_2_ group, a total of 95 DEGs were found in the MH group, including 59 downregulated and 36 upregulated genes (Figure 4B). A heatmap of DEGs is shown in Figure 4C, which displays a contrasting gene expression profile between different groups.

### 3.5. Functional Enrichment of DEGs

**Figure 5:**
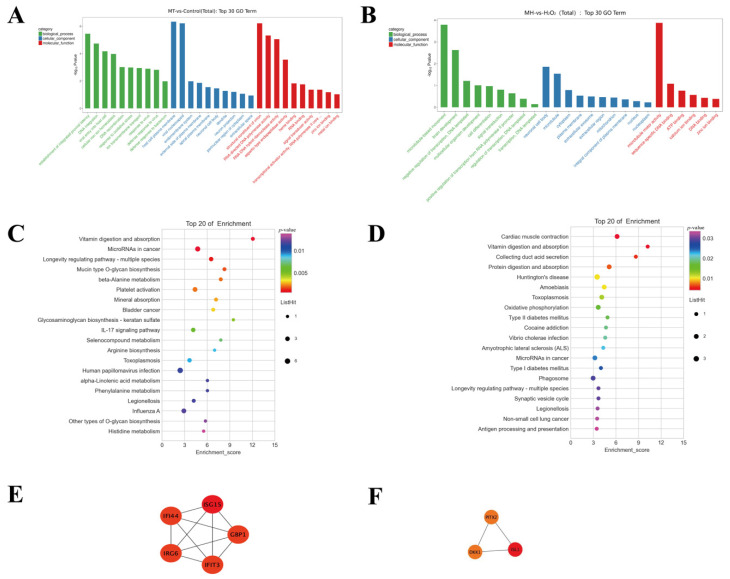
KEGG pathway enrichment, GO functional annotation and PPI network analysis for DEGs. (**A**) the top 30 enriched GO terms (MT vs. CON); (**B**)the top 30 enriched GO terms (MH vs. H_2_O_2_); The *y*-axis represents enrichment factor. The *x*-axis represents GO terms. The pathways with numbers of differential genes greater than 2 were screened. (**C**) the top 20 KEGG enriched pathways (MT vs. CON); (**D**) the top 20 KEGG enriched pathways (MH vs. H_2_O_2_); The *y*-axis The y-axis corresponds to the KEGG Pathway. The *x*-axis shows the enrichment factor. The color of the dot represents the *p*-value and the size of the dot represents the number of DEGs mapped to the reference pathways. (**E**) Module 1 (MT vs. CON). (**F**) Module 2 (MH vs. H_2_O_2_). The color indicate in red (high score) and yellow (low score).

To further analyze the function of DEGs, we performed a pathway analysis. As shown in Figure 5, the GO analysis showed that the DEGs were significantly enriched into the terms, including “establishment of integrated proviral latency” in the biological process (BP) category, “host cell plasma membrane” in the cellular component (CC) category, and “structural constituent of virion” in the molecular function (MF) category (MT vs. CON) (Table S1). Furthermore, “microtubule-based movement” was identified in the BP category, “neuronal cell body” was identified in the CC category, and “microtubule motor activity” was identified in the MF category between the MH and H_2_O_2_ groups (Table S2). In addition, the KEGG pathway enrichment analysis showed that upregulated DEGs in MT pretreatment were significantly enriched in the “Wnt signaling pathway”, “TGF-beta signaling pathway”, and “mTOR signaling pathway” and so on (Figure 5C,D).

To further screen for hub genes, a network of the DEGs was obtained using STRING, MCODE and CytoHubba to identify the modules and hub genes in Cytoscape. As shown in Tables S3 and S4, the hub genes were identified by CytoHubba plug-ins in the groups (MT vs. CON) and groups (MH vs. H_2_O_2_), respectively. Furthermore, the most significant 1 modules were filtered using the MCODE (Figure 5C,D).

### 3.6. qRT–PCR Validation

**Figure 6:**
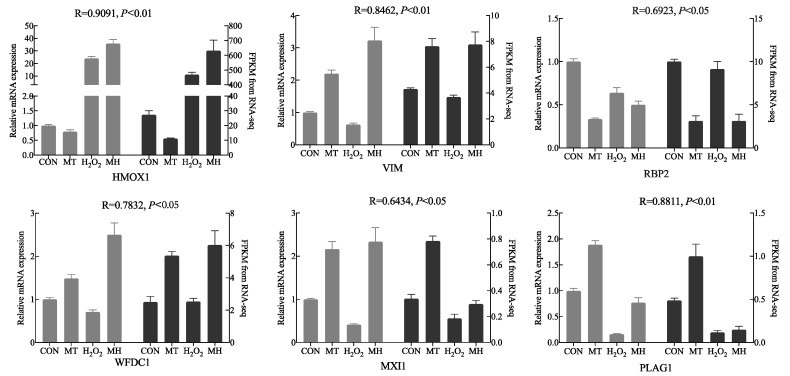
Comparison of the gene expression levels of RNA-seq with real-time PCR. The right axis represents the expression levels determined by RNA-seq in FPKM units, and the left axis represents gene expression levels determined by real-time PCR. Bars represent the mean ± SEM of three samples. The black column indicates the FPKM value; the grey column indicates the real-time PCR using *β-actin* as a reference gene.

To validate the accuracy of the RNA-seq data and detect DEGs, we screened six DEGs (*HMOX1*, *VIM*, *RBP2*, *WFDC1*, *MXI1* and *PLAG1*) for qRT-PCR analysis. The same trends in gene expression were detected in the data obtained by RNA-seq and qPCR, suggesting that RNA-seq accurately quantified cell gene expression in the CON, MT, H_2_O_2_ and MH groups (Figure 6). Notably, the significant positive correlation between RNA sequencing data and a qRT-PCR analysis supported the results found.

### 3.7. Effects of MT Pretreatment on H_2_O_2_-Induced Protein Expression in PTr2 Cells

**Figure 7:**
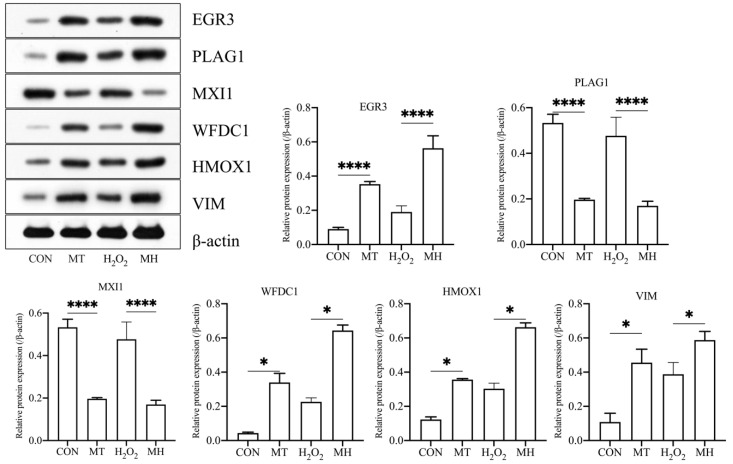
MT protects PTr2 cells from H_2_O_2_-induced oxidative stress by inducing proteins expression. Western blot analysis were performed to detect the protein levels in different group. The data are expressed as the means ± SEM from at least 3 separate experiments. *p* < 0.05 was considered to be statistically significant. *p* > 0.05 (ns), *p* < 0.05 (*), *p* < 0.0001 (****).

To investigate the effect of MT on H_2_O_2_-mediated oxidative stress in PTr2 cells, we selected *EGR3*, *PLAG1*, *MXI1*, *WFDC1*, *HMOX1* and *VIM* for the Western blot analysis. As shown in Figure 7, the *EGR3*, *HMOX1*, *VIM* and *WFDC1* proteins were significantly upregulated in the MT group compared with the CON group and significantly were upregulated in the MH group compared with the H_2_O_2_ group. However, the expression levels of the *PLAG1* and *MXI1* were significantly lower in the MT group than in the CON group, and significantly lower in the MH group than in the H_2_O_2_ group. The corresponding differentially expressed genes are listed in Table 1.

## 4. Discussion

Oxidative stress in the placenta can lead to reproductive dysfunction, and can be transmitted to offspring [39,40]. Some antioxidants have been shown to reduce oxidative damage in animals, but this remains to be further explored [41]. Therefore, in this study, the protective effects of MT on oxidative stress were investigated.

PTr2 cells are significant cell types of the placenta and play important roles in fetal nutrition and growth throughout development [42]. However, overproduction of ROS may lead to placental oxidative stress and cell apoptosis [11,43,44]. We observed that pretreatment with 500 μM MT ameliorated the H_2_O_2_-induced reduction in PTr2 cell viability and significantly decreased ROS production. These results indicated that MT pretreatment exhibited antioxidant activity, consistent with previous studies showing that MT has various positive anti-apoptotic and antioxidative stress effects during pre- and postimplantation development of cloned mouse embryos [45,46]. Furthermore, we found that MT (500 μM) pretreatment significantly alleviated H_2_O_2_-induced cell apoptosis, suggesting that MT might alleviate H_2_O_2_-induced oxidative damage by reducing ROS levels and protecting cells against apoptosis.

To filter candidate genes involved in the regulation of the oxidative stress response, a transcriptome analysis was further performed in cultured PTr2 cells with different treatments. The GO enrichment analysis of upregulated DEGs (CON vs. MT) showed that “response to oxidative stress” (GO: 0006979) was significantly enriched. The results indicated that DEGs involved in “positive regulation of the Wnt signaling pathway” were upregulated in the MH group.

The KEGG pathway enrichment analysis showed that the Wnt signaling pathway, “TGF-beta signaling pathway” and “mTOR signaling pathways” were significantly enriched for DEGs between the MH and H_2_O_2_ groups. The Wnt signaling pathway has been reported to be redox-sensitive and regulated by oxidative stress [47,48,49]. In the present study, DEGs were significantly enriched in the Wnt signaling pathway, suggesting that MT may play a protective role in H_2_O_2_-induced oxidative stress in PTr2 cells by regulating the Wnt signaling pathway. In addition, the TGF-beta signaling pathway is also an effective signaling pathway involved in the acceleration of oxidative stress and apoptosis [50]. Previous studies have shown that long-chain fatty acids (LCFAs) facilitate hepatocyte activation by upregulating oxidative stress through TGF-β signaling pathway-related genes [51]. Compared with H_2_O_2_-treated cells, *PITX2* was identified as an upregulated DEG in MT-treated PTr2 cells, and it was demonstrated to be significantly enriched in the TGF-beta signaling pathway. *PITX2* is a pivotal component of both the TGF-beta and Wnt/beta-catenin signaling pathways, which play an important role in TGF-beta signaling pathway [52]. Together, these results suggest that MT alleviates H_2_O_2_-induced oxidative stress by activating the TGF-beta signaling pathway. The mTOR signaling pathway is also related to oxidative stress or antioxidant capacity [53,54]. In this study, *ATP6V1C2* was upregulated in MT-treated PTr2 cells and was enriched in the mTOR signaling pathway. Similar results have been described indicating that melatonin alleviates high glucose-induced apoptosis in Schwann cells through the mTOR and Wnt signaling pathways [55].

Our study suggests that MT may serve as a protective agent against oxidative stress-induced PTr2 cell apoptosis. According to the results, MT (500 μM) pretreatment significantly increases the expression of *HMOX1*, *VIM* and *EGR3* at both the mRNA and protein levels in PTr2 cells. Previous studies have reported that *HMOX1*, *VIM* and *EGR3* can protect cells against oxidative stress and play roles in cellular antioxidant defense [56,57,58,59]. *HMOX1* is a commonly used marker of oxidative stress, and its increased expression is associated with resistance to oxidant-induced apoptosis as an adaptation response [60,61]. In addition, the activation of *HMOX1* is involved in heme catabolism and induced by oxidative stress [62]. In this study, MT-pretreated cells showed higher *HMOX1* expression levels than the H_2_O_2_ group, suggesting that MT (500 μM) may alleviate oxidative stress by upregulating the expression of *HMOX1*.

Previously, *VIM* has been shown to effectively improve the decreased epithelial-to-mesenchymal transition (EMT) capacity of placental trophoblast cells caused by hypoxia [63]. *VIM* is significantly increased in response to increased ROS under oxidative stress conditions in H_2_O_2_-treated renal cell lines [64]. Loss of *VIM* function in mice results in defects in the response and adaptation to oxidative damage [65]. Here we showed that MT (500 μM) significantly reduced H_2_O_2_-induced apoptosis in vitro, which may be involved in upregulating the expression of *VIM*.

*EGR3* is a member of the EGR family transcription factors that regulate cell responses to proliferation, survival and apoptosis [66,67]. In this work, we found that MT (500 μM) pretreatment significantly increased *EGR3* expression and was associated with increased PTr2 cell proliferation and decreased apoptosis compared with the H_2_O_2_ group in vitro. Therefore, MT (500 μM) may affect the expression of antioxidant-related proteins (such as *HMOX1*, *VIM*, *EGR3*) to alleviate oxidative stress induced by H_2_O_2_.

Additionally, we prioritized the module hub genes *ISG15* and *ISL1* by CytoHubba in groups (MT vs. CON) and groups (MH vs. H_2_O_2_), respectively. *ISG15* is a stress response gene that may act as a contributor to tumor suppressors and inflammatory responses [68]. Previous studies have shown that *ISG15* can reach high levels in response to oxidative stress [69]. Moreover, *ISL1* was downregulated to protect cells from apoptosis under oxidative stress conditions [70]. Interestingly, our study showed that *ISL1* gene expression was lower in the MH group than in the H_2_O_2_ group, suggesting that MT might alleviate the oxidative stress by downregulating *ISL1* expression.

## 5. Conclusions

In summary, MT effectively protects H_2_O_2_-treated PTr2 cells against oxidative stress by decreasing intracellular ROS generation and cell apoptosis and regulating the expression of antioxidant-related genes. MT as an antioxidant candidate might protect PTr2 cells from excessive oxidative stress during pregnancy. These results are expected to provide a theoretical basis for the development of the clinical application of MT during pregnancy and provide guidance for developing effective nutritional strategies to improve sow health.

## Figures and Tables

**Table 1:** RT-qPCR measurement of differential gene expression.

Gene Name	CON	MT	*p*-Value	H_2_O_2_	MH	*p*-Value
*HMOX1*	1 ± 0.0613	0.793 ± 0.109	0.046	24.073 ± 2.338	35.906 ± 5.714	0.029
*VIM*	1 ± 0.057	2.204 ± 0.187	0.005	0.629 ± 0.077	3.228 ± 0.710	0.003
*WFDC1*	1 ± 0.078	1.488 ± 0.151	0.008	0.711 ± 0.083	2.502 ± 0.469	0.003
*MXI1*	1 ± 0.0416	2.164 ± 0.304	0.003	0.417 ± 0.034	2.337 ± 0.559	0.004
*PLAG1*	0.999 ± 0.078	1.887 ± 0.134	0.001	0.163 ± 0.015	0.773 ± 0.161	0.003
*EGR3*	1 ± 0.106	1.948 ± 0.366	0.013	1.868 ± 0.134	42.606 ± 4.535	0.004

The RT-qPCR results are expressed as the mean ± SEM of at least three independent experiments.

## Data Availability

The raw reads were deposited to Sequence Read Archive (SRA) database (PRJNA820162 for RNA-seq).

## Supplementary Materials

The following supporting information can be downloaded at: https://www.mdpi.com/article/10.3390/antiox11061047/s1. Table S1: GO enrichment analysis of DEGs (DEGs in MT group vs. CON group); Table S2: GO enrichment analysis of DEGs (DEGs in MH group vs. H_2_O_2_ group); Table S3 Hub genes ranked by the Degree method in CytoHubba (MT VS. CON); Table S4: Hub genes ranked by the Degree method in CytoHubba (MH VS. H_2_O_2_).
