# Supplementary material for: Melatonin Alleviates Oxidative Stress Induced by H2O2 in Porcine Trophectoderm Cells
Source: Antioxidants (Basel). 2022 May 25;11(6):1047. doi: 10.3390/antiox11061047 (PMC9219737; doi:10.3390/antiox11061047)
Supplement: Supplementary file 1 [file antioxidants-11-01047-s001.zip › Supplementary Materials/Table S3.pdf]

Table S3. Hub genes ranked by the Degree method in cytoHubb (MT VS CON).

**Catelogy**	**Rank methods in cytoHubba**					
	**MCC**	**MNC**	**Degree**	**EPC**	**Closeness**	Radiality
Gene symbol top 15	ISG15	ISG15	ISG15	ISG15	MYC	MYC
	IRG6	IRG6	MYC	GBP1	ISG15	ISG15
	IFIT3	IFIT3	IRG6	IFIT3	GBP1	THBS1
	IFI44	IFI44	IFIT3	IFI44	IFIT3	MXI1
	GBP1	GBP1	IFI44	MYC	IFI44	FOSL1
	MYC	FCGR2B	GBP1	IRG6	IRG6	GBP1
	THBS1	SPN	FCGR2B	THBS1	THBS1	IFIT3
	FCGR2B	CD5	THBS1	MXI1	MXI1	IFI44
	SLC5A8	MYC	SPN	FOSL1	FOSL1	IRG6
	PRKAG3	THBS1	CD5	ZFAND2A	MMP16	MMP16
	SRM	SLC5A8	SLC5A8	MMP16	ZFAND2A	ZFAND2A
	MXI1	PRKAG3	PRKAG3	ITGA2B	ITGA2B	ITGA2B
	GCNT3	SRM	SRM	SEMA5A	SEMA5A	SEMA5A
	MUC16	MXI1	MXI1	FCGR2B	PSD	PSD
	FOSL1	GCNT3	GCNT3	PSD	SNX32	SNX32
