# Supplementary material for: Melatonin Alleviates Oxidative Stress Induced by H2O2 in Porcine Trophectoderm Cells
Source: Antioxidants (Basel). 2022 May 25;11(6):1047. doi: 10.3390/antiox11061047 (PMC9219737; doi:10.3390/antiox11061047)
Supplement: Supplementary file 1 [file antioxidants-11-01047-s001.zip › Supplementary Materials/Table S4.pdf]

Table S4. Hub genes ranked by the Degree method in cytoHubb (MH VS H_2O_2).

**Catelogy**	**Rank methods in cytoHubba**					
	**MCC**	**MNC**	**Degree**	**EPC**	**Closeness**	Radiality
Gene symbol top 15	ISL1	ISL1	ISL1	ISL1	VIM	SNAI2
	VIM	PITX2	VIM	VIM	ISL1	VIM
	SNAI2	DKK1	PITX2	SNAI2	SNAI2	ISL1
	MSRA	VIM	DKK1	PITX2	COL3A1	COL3A1
	FAM213B	SNAI2	SNAI2	DKK1	PITX2	PRPH
	PITX2	MSRA	MSRA	COL3A1	DKK1	PITX2
	DKK1	FAM213B	FAM213B	FAM213B	PRPH	DKK1
	COL3A1	COL3A1	COL3A1	MSRA	COL16A1	COL16A1
	PRPH	PRPH	PRPH	PRPH	FAM213B	MSRA
	STK31	STK31	STK31	COL16A1	MSRA	FAM213B
	SLC22A20	SLC22A20	SLC22A20	DNAJB1	DNAJB1	STK31
	LAX1	LAX1	LAX1	STK31	STK31	DNAJB1
	IL1R2	IL1R2	IL1R2	PRR18	PRR18	SLC22A20
	IL18R1	IL18R1	IL18R1	FAM171B	FAM171B	LAX1
	NCKAP5	NCKAP5	NCKAP5	IL1R2	IL1R2	IL1R2
